# Ovarian Stimulation for In Vitro Fertilization and Reproductive Outcome after Surgical Treatment of Endometriosis Compared with Tubal Factor Infertility

**DOI:** 10.3390/clinpract14010001

**Published:** 2023-12-20

**Authors:** Elena-Silvia Nadă, Cătălin Bogdan Coroleucă, Ciprian Andrei Coroleucă, Elvira Brătilă

**Affiliations:** 1 Department of Obstetrics and Gynecology, “Carol Davila” University of Medicine and Pharmacy, 020021 Bucharest, Romania; ciprian.coroleuca@umfcd.ro (C.A.C.); elvira.bratila@umfcd.ro (E.B.); 2 Department of Obstetrics and Gynecology, “Prof. Dr. Panait Sîrbu” Clinical Hospital of Obstetrics and Gynecology, 060251 Bucharest, Romania; ccoroleuca@yahoo.com

**Keywords:** endometrioma, gonadotropin, infertility, ovarian stimulation, pregnancy rate, IVF

## Abstract

Endometriosis is a common cause of infertility among reproductive-age women. A low ovarian reserve is associated with the presence of endometriotic cysts, and this is accentuated even more after surgery. Patients with a history of endometrioma are a special category of poor ovarian reserve requiring in vitro fertilization (IVF). The aim of this retrospective study was to evaluate the characteristics and outcome of ovarian stimulation and embryo transfer in women with a history of ovarian surgery for endometrioma compared with a control group with tubal factor infertility. A total of 146 patients had previous laparoscopic cystectomy for endometrioma (group A) and their IVF results were compared with 136 patients with documented tubal obstruction (group B). In both groups, the most frequently used ovarian stimulation protocol was the short antagonist in 84.24% versus 80.88%. The number of stimulation days was between 6 and 15 days in the two groups with a mean value of 12.76 days in group A and 9.47 days in group B. The clinical pregnancy rate was 26.77% in the endometrioma group and 39.68% in the tubal obstruction group. Patients with a history of endometrioma are less likely to conceive than those with tubal obstruction despite having similar ovarian reserve and stimulation results.

## 1. Introduction

Endometriosis is a chronic, benign disease defined by the presence of endometrial glands and stroma outside of the uterine cavity. It is often a multifocal condition with a slow but progressive course in the absence of adequate management. Globally, the incidence of this pathology is approximately 10–15% in women of reproductive age, reaching up to 30–50% in patients with chronic pelvic pain or infertility [1,2,3].

The most common symptoms associated with endometriosis are linked with pain—dysmenorrhea, non-cyclic pelvic pain and dyspareunia, but the intensity of painful symptoms is not correlated with the stage of the disease. Studies have shown that almost 20–25% of endometriosis cases are asymptomatic (silent endometriosis), thus allowing the disease to progress in the absence of a diagnosis with a direct impact on the ovarian reserve and in addition diminishing the chances of conception even with IVF [4].

Laparoscopy was until recently considered the gold standard in the diagnosis of endometriosis due to the ability to directly visualize the lesions in the peritoneal cavity with subsequent histopathological confirmation. The European Society of Human Reproduction and Embryology (ESHRE) 2022 guideline regarding endometriosis no longer considers laparoscopy as the gold standard for the diagnosis of endometriosis and recommends that it should be used only in patients with normal imaging or in cases with unsuccessful empirical treatment [5].

Endometriomas are the most common and easily recognized ultrasound marker of endometriosis. Ovarian endometriomas are considered “the tip of the iceberg” in terms of endometriotic lesions, a marker of a more extensive disease when it comes to mapping all endometriotic lesions. According to the ESHRE guideline, clinicians are encouraged to use ultrasound or magnetic resonance imaging in the diagnosis of endometriosis, but they should be aware that a negative examination does not rule out the disease, especially when it comes to superficial peritoneal implants [5,6]. Transvaginal ultrasound has 73% sensitivity and 94% specificity for the diagnostic of ovarian endometrioma, while MRI has 95% sensitivity and 91% specificity [7,8]. If an endometrioma is identified, then complete evaluation of all possible endometriotic lesions is mandatory to stage the disease and to establish the optimal management. Endometriotic cyst excision prior to IVF should not be performed solely to improve pregnancy rates because of the detrimental effect on the ovarian reserve. The basic principle in endometriosis surgery is based on the radicality of exeresis, that is, the complete excision of all endometriotic lesions with minimal ovarian damage in a single surgical intervention, “one stop shop surgery”, in a center of expertise [5,9,10].

Endometriomas interfere with the development of ovarian follicles, not only in a natural cycle, but also in the case of ovarian stimulation during the in vitro fertilization (IVF) procedure, where a decrease in the number of retrieved oocytes is observed, leading to a suboptimal response. It has been shown that while surgery increases the chances of a spontaneous pregnancy, it is still associated with a subsequent decrease in the ovarian reserve. In the context of endometriosis, a decreased ovarian reserve has a significantly impaired fertility prognosis compared with a decreased ovarian reserve attributed to other causes. Several hypotheses have been described to underlie the low ovarian reserve associated with endometrioma: severe local inflammation, oxidative stress due to increased production of reactive oxygen species leading to fibrosis, compromised ovarian vascularization due to excessive electrocoagulation and accidental excision of an area of normal ovarian tissue during cystectomy [9,11,12,13,14].

Laparoscopic cystectomy may be useful before IVF procedures, because in the presence of endometriomas, higher doses of gonadotropins may be required for ovarian stimulation, and also, the presence of large endometriomas may impair oocyte pick-up. In addition, there is a higher risk of endometrioma perforation during oocyte pick-up with the additional alteration of oocyte quality. A wait-and-see approach is recommended in patients with a low ovarian reserve, anti-Mullerian hormone (AMH) < 0.5 ng/mL, because there is an increased risk of failure to conceive even with IVF. But in the case of bulky endometrioma in a patient with an already low ovarian reserve, ultrasound-guided drainage associated with ethanol sclerotherapy may be considered before ovarian stimulation [5,15,16].

The management of infertility associated with endometriosis can be schematically divided into two directions: surgical intervention followed by assisted reproduction techniques or only assisted reproduction techniques. The surgical treatment and postoperative management are tailored according to the extent of the endometriotic lesions, taking into account the presence of other determining factors of infertility, the patient’s age, her desire to procreate in the near or distant future, previous ovarian surgery, ovarian reserve and associated male infertility [6].

Most often, women with a history of ovarian surgery due to endometrioma turn to IVF in order to conceive. Two ovarian stimulation protocols are equally recommended for patients with endometriosis and a low ovarian reserve and are chosen according to patient characteristics and physician experience—the short GnRH (gonadotropin releasing hormone) antagonist (short protocol) and long GnRH agonist (long protocol) but also the luteal phase stimulation and the dual stim protocol have gained an increased popularity in this category of patients.

Regarding the use of gonadotropin for controlled ovarian stimulation, recombinant FSH, follicle-stimulating hormone (follitropin alfa, follitropin beta, follitropin delta); purified FSH (p-FSH); a combination of recombinant FSH with recombinant LH, luteinizing hormone (follitropin alfa with lutropin alfa); long-acting recombinant FSH (corifollitropin alfa); and human menopausal gonadotropin (menotropin) are equally recommended and considered safe in terms of efficacy. Other substances such as aromatase inhibitors (letrozole) or clomiphene citrate are not recommended as a substitute for gonadotropins in controlled ovarian hyperstimulation; they are currently used in mild stimulation for intrauterine insemination [17].

The aim of this study is to evaluate the clinical and paraclinical characteristics of patients with endometriosis and infertility and the outcome of ovarian stimulation and embryo transfer associated with a successful reproductive outcome in women with a history of ovarian surgery for endometrioma compared to a control group of patients with tubal factor infertility.

## 2. Materials and Methods

We conducted a retrospective study among women diagnosed with infertility who underwent assisted reproduction procedures in the Assisted Human Reproduction Department of the “Prof Dr Panait Sîrbu” Clinical Hospital of Obstetrics and Gynecology in Bucharest, a center with 25 years of experience in the field. The study was carried out between January 2019 and December 2022.

Inclusion criteria included the following: age 18–40 years, laparoscopic ovarian cystectomy for endometrioma (group A), tubal obstruction documented by hysterosalpingography or laparoscopy (group B). Exclusion criteria included the following: teratospermia on semen analysis, IVF with donated oocytes, sperm donation, embryo donation, history of major diseases with impact on fertility (cancer, cardiovascular or psychiatric disease). The statistical analysis was performed using Microsoft Excel 2021.

The initial assessment of patients included complete blood count, blood type, Rh factor, blood tests for chronic or active infections (hepatitis B, hepatitis C, HIV, VDRL, TPHA), TORCH test, tests for high-risk thrombophilia, urinalysis, Pap test, day 2–3 hormonal assay (FSH, LH, estradiol, TSH, freeT4, ATPO, AMH), cardiac evaluation with EKG and breast ultrasound. Before the ovarian stimulation process, they were also screened for vaginal infections, Chlamydia trachomatis, Neisseria gonorrhoeae, *Mycoplasma* spp. and *Ureaplasma* spp. in order to perform hysteroscopy and tubal patency test. In our human reproduction department, all women perform hysteroscopy and tubal patency test before embryo transfer in order to document tubal obstruction or hydrosalpinx. If the latter is diagnosed, then tubal ligation or salpingectomy is performed in order to improve pregnancy rates.

Ovarian stimulation was performed using 3 IVF protocols: short GnRH antagonist, long GnRH agonist and dual stim. The short antagonist protocol was started on the 2nd or 3rd day of the menstrual cycle after ultrasound examination with the administration of gonadotropins. Patients were monitored with regular transvaginal ultrasound examinations on day 5 (when the GnRH antagonist is introduced), day 8 of stimulation and every 2–3 days thereafter to decide the timing of ovulation triggering. Measurements of hormone levels (LH, estradiol, progesterone) were also performed before ovulation triggering. Final oocyte maturation is decided when there are more than 3 ovarian follicles >17 mm in diameter, and in this protocol, we used hCG (human chorionic gonadotropin) trigger alone or dual trigger (hCG + GnRH agonist). The long GnRH agonist protocol was started on the 21st day of the previous menstrual cycle with the administration of the GnRH agonist (a single dose of 3.75 mg or 0.1 mg daily). After 14 days, ovarian suppression was certified with a serum estradiol measurement (<50 pg/mL) and transvaginal ultrasound (endometrium < 5 mm and ovarian follicles < 10 mm) to start the ovarian stimulation. Patients were then monitored regularly on the 5th, 8th day of stimulation and every 2–3 days thereafter to decide the timing of ovulation, with hCG alone. The decision to use the dual stim protocol was made in poor responder patients or with low ovarian reserve. Dual stim combines ovarian stimulation in the follicular phase with ovarian stimulation in the luteal phase. The difference is in the oocyte triggering, which is always carried out with a GnRH agonist in the first stimulation cycle, and 5 days after the oocyte retrieval, a new cycle of ovarian stimulation with gonadotropins begins. Oocyte retrieval is scheduled 35–36 h after the trigger is administered and is performed under anesthesia.

Stimulation protocols, types and doses of gonadotropins were chosen according to clinical and paraclinical characteristics (age; AMH; antral follicle count; BMI—body/mass index; records of previous stimulation protocols) and based on the experience of the fertility specialist. The daily dose did not exceed 300 UI no matter the patient’s BMI or ovarian reserve.

Fresh or frozen single embryo transfer was performed with day 3 or day 5 embryos. Luteal phase support was offered to all patients in a personalized manner with progesterone (injectable, vaginal in all cases), estradiol, low dose aspirin, low-molecular-weight heparin, corticosteroids, prenatal vitamin supplements.

Determination of serum beta—human chorionic gonadotropin (hCG)—was performed 10 days after embryo transfer to confirm the biochemical pregnancy, and transvaginal ultrasound was performed after 5 weeks to confirm the clinical pregnancy.

## 3. Results

A total of 1107 patients underwent IVF procedures between January 2019 and December 2022 in the Human Assisted Reproduction Department of the “Prof Dr Panait Sîrbu” Clinical Hospital of Obstetrics and Gynecology. In total, 226 patients were diagnosed with endometriosis, of whom 146 patients met the inclusion and exclusion criteria and had previous laparoscopic cystectomy for endometrioma (group A) and were compared with 136 patients with documented tubal obstruction (group B).

The clinical characteristics, ovarian stimulation and reproductive outcome of the two studied groups are found in Table 1.

The ovarian stimulation protocol, the gonadotropin type and dose were chosen by the fertility specialists according to age, AMH, antral follicle count and BMI. The types and doses of gonadotropin were not changed during the stimulation period, as this practice is not associated with a better outcome in terms of the number of retrieved oocytes or pregnancy rate.

The number of stimulation days was identical between the two groups between 6 and 15 days, but more than 10 days of gonadotropin use was required in 80.13% of patients with endometrioma and only in 29.41% of patients with tubal obstruction.

**Figure 1:**
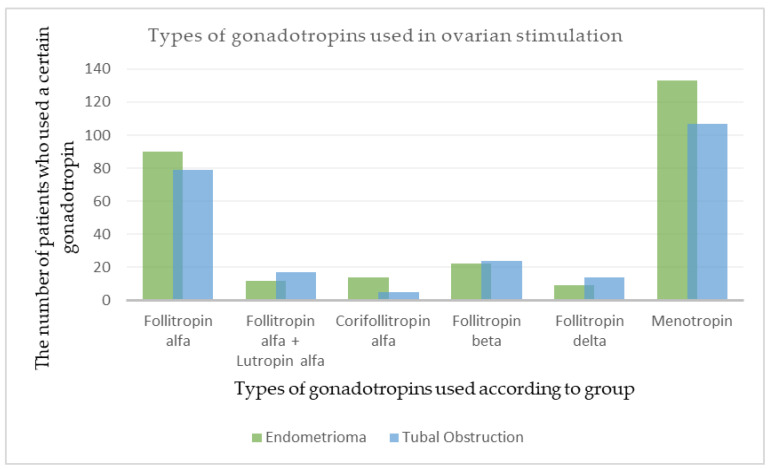
Types of gonadotropins used in ovarian stimulation.

The gonadotropin dose was between 150 and 300 UI daily. The most-used gonadotropin was follitropin alfa in both groups (61.64% in group A and 58.08% in group B). The least-used were follitropin delta in the endometrioma group (6.16%) and corifollitropin alfa in the tubal obstruction group (3.67%). Menotropin was not used as a single medication for stimulation, but only in addition to follitropin in 133 patients (91.09%) from the endometrioma group and in 107 patients (78.67%) in the tubal obstruction group. The addition of menotropin to the stimulation protocol was added in a personalized manner, based on the experience of the fertility specialists and the records of previous stimulation results for that patient. (Figure 1).

There were no registered cases of cycle cancellation due to a suboptimal response to gonadotropins or ovarian hyperstimulation syndrome, regardless of the type of protocol used or the ovulation induction medication.

IVF-ICSI (intracytoplasmic sperm injection) was performed for the most part in both groups, 74.10% and 53.12%, respectively. The cancellation rate due to a lack of mature oocytes was 4.79% in group A (7 patients) and slightly higher in group B at 5.88% (8 patients). The cancellation rate due to fertilization failure was 4.10% in group A and only 1.47% in group B in the absence of an altered semen analysis.

Only single embryo transfer was performed with a fresh or frozen day 3 embryo or a blastocyst. The number of embryos obtained was the same in the two groups, between 0 and 9, and the average number of embryos was 3.93 in group A and 3.37 in group B. Nevertheless, 13 patients from group A and 10 from group B were not able to obtain any embryos. The maximum number of embryos was obtained from four patients in group A and from only one in group B.

Fresh embryo transfer was mainly performed in group B (61.11%), while frozen embryo transfer was mostly performed in group A (64.56%).

The determination of serum beta-hCG was obtained 10 days after embryo transfer. The overall biochemical pregnancy rate was 42.51% in the endometrioma group and 46.82% in the tubal obstruction group. The clinical pregnancy rate was 26.77% in the endometrioma group and 39.68% in the tubal obstruction group.

Considering that a high BMI is a risk factor for infertility, we analyzed the two groups from this perspective. In the endometrioma group, we mostly had normal-weight patients (68.49%), but there were also 20.54% underweight patients, 10.27% overweight patients and one obese patient with a BMI of 30.11 kg/m^2^. The clinical pregnancy rate in the overweight category was 26.66%, which in this case coincided with the clinical pregnancy rate of the entire group (26.77%), and the only obese patient achieved pregnancy. In the tubal obstruction group, we had 50% overweight patients and 11.02% obese patients with 41.65% and 35.06% pregnancy rates.

The clinical characteristics of the patients who achieved pregnancy were further analyzed in comparison with the whole group and are listed in Table 2.

Analyzing data from patients who achieved clinical pregnancy, it turned out that AMH is a detrimental factor in both groups—most patients with AMH higher than 3 ng/mL had a positive outcome (57.14% and 60%). The category of patients with AMH below 1 ng/mL had the lowest pregnancy rate in both groups (18.96% and 23.61%). Regarding the ovarian stimulation protocol, dual stim had the most favorable result (40% and 60%, respectively, of the patients with the dual stim protocol got pregnant).

The correlation between the type of gonadotropins used in relation to pregnancy rates showed significant differences. In the endometrioma group, administration of corifollitropin alfa was associated with the highest pregnancy rate (57.14%) and, surprisingly, the lowest (20%) in patients with tubal obstruction. The results of our study showed a better reproductive outcome when corifollitropin alfa was used in patients with endometriosis than in patients with tubal factor infertility, but the number of cases was insufficient to draw a conclusion. Corifollitropin was used in only 16 patients in our group. The higher pregnancy rate associated with this gonadotropin may be related to the fact that the patients who achieved pregnancy were all under 35 years of age, normal weight, non-smokers, had an average AMH value of 1.54 ng/mL, and all had embryo transfers with a blastocyst.

**Figure 2:**
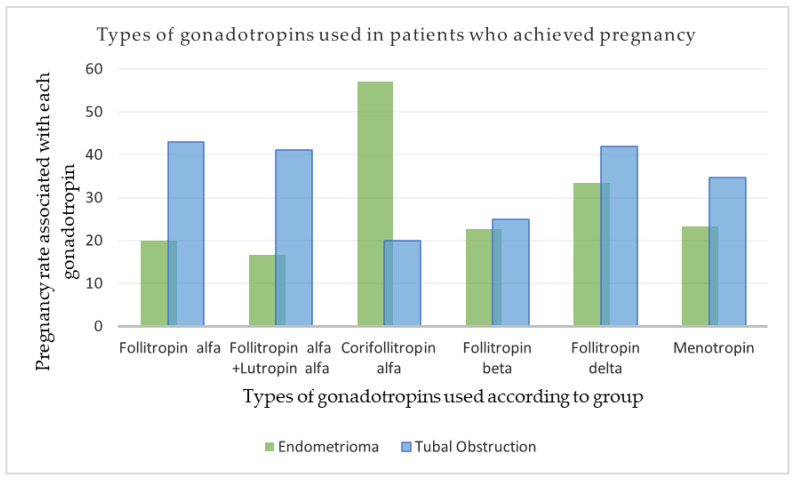
Types of gonadotropins used in patients who achieved pregnancy in relation to the whole group.

A significant difference in pregnancy rate was observed with follitropin alfa in the endometrioma group (20%) and the control group (43.03%). Similar results between the two groups in terms of pregnancy rate were revealed only after follitropin beta administration (22.72% vs. 25%) (Figure 2).

## 4. Discussion

Endometriosis is a common chronic inflammatory disease that affects women mainly in their reproductive years. It is a frequent cause of infertility due to the progressive decrease in the ovarian reserve, as well as due to the altered anatomy, impaired oocyte quality and hormonal imbalance. Despite being common, it is still one of the most underdiagnosed conditions in gynecology, with a delay of up to 8 years from the onset of symptoms to diagnosis [18,19].

Endometriosis is a benign disease capable of altering the overall quality of life, with social, professional and especially family planning implications. Nowadays, there is a tendency to postpone the age at which a woman is planning to conceive, which is pushed even further when dealing with endometriosis-associated infertility. Available data from 2021 show that the highest average age at birth in Europe was 32.2 years and was registered in Luxembourg and Ireland, while in Romania, it was 28.1 years [20]. The results of our study support this trend, with the average age in our groups being 34 and 37 years. This advanced childbearing age can be attributed both to social reasons and to diagnostic delays in infertility associated with endometriosis or tubal obstruction.

A significant difference is noted in the endometrioma group when correlating the patients’ age with the pregnancy rate. In total, 32.85% of patients under 35 years got pregnant compared to only 14.47% in the over 35 years category, and the same difference was observed in the tubal pathology group (60.37% vs. 21.68%). This supports the idea that age is a detrimental factor for pregnancy rate, and in addition, the presence of endometrioma is a cause of the natural decrease in the ability to achieve pregnancy. A 2018 study in a tertiary center in Turkey of 40 patients with endometrioma reported a 26.4% decrease in the ovarian reserve in just 6 months in the absence of surgery, as opposed to only a 7.4% decrease in the case of women without endometriosis [21].

Most studies and meta-analyses support the idea that endometriomas and especially ovarian surgery for endometriomas produce a decrease in ovarian reserve. A 2022 study reports a decrease in AMH levels from an average of 3.77 ng/mL preoperatively to 1.72 ng/mL at 12 months postoperatively [22,23,24,25]. This decrease in AMH levels has a crucial influence in choosing the appropriate ovarian stimulation protocol, type and dose of gonadotropin in order to maximize IVF success. In our study, the mean AMH value was similar between the two groups, 1.54 ng/mL versus 1.47 ng/mL, despite ovarian surgery in the first group, which theoretically may lead to a favorable outcome as far as fertility is concerned. A major difference in the ovarian reserve was not registered in our study, and all patients were operated in an Endometriosis Center of Excellence with surgical expertise.

IVF should be offered to patients with endometriosis-associated infertility, especially if there is male or tubal factor infertility, low ovarian reserve, low EFI score and failure to conceive naturally. Regarding the type of ovarian stimulation protocol, either short GnRH antagonist or long GnRH agonist can be used depending on the clinical characteristics of the patient, as numerous studies show no difference in the implantation rate and clinical pregnancy rate [5,26]. In our study, in the majority of cases, we used the short GnRH antagonist protocol due to its advantages: a shorter stimulation period, lower inhibition, lower doses of gonadotropins, increased patient comfort and minimal chance of hyperstimulation syndrome. However, similar pregnancy rates were obtained with short and long protocol in the endometrioma group. Similar results in terms of implantation rate and pregnancy rate were encountered in a randomized trial including 246 patients with endometrioma when taking into account the two protocols [27]. Despite being used the least, the dual stim protocol had the best outcome in terms of pregnancy rate in both groups, but due to the small number of patients who followed this protocol, we are not able to draw a conclusion. A significant difference consistent with other studies was noted regarding the total stimulation period, that is, 80.13% of patients with endometrioma required more than 10 days of gonadotropins, as opposed to only 29.41% of patients with tubal obstruction [5,27,28,29].

Choosing the appropriate type and dose of gonadotropin from the wide range available nowadays is probably the most important factor in IVF outcome, as it is one of the few modifiable factors. In our study, the use of corifollitropin alfa showed better results in patients with endometrioma in terms of pregnancy rate compared to other gonadotropins. A single dose of this long-acting rFSH is administered at the beginning of the stimulation protocol and is sufficient for 7 days of stimulation, resulting in a reduction in the total number of injections, thus increasing patient comfort. Its serum half-life is 2–3 times longer than other follitropins due to lower hepatic and renal excretion. Several meta-analyses demonstrate the effectiveness of corifollitropin alfa in terms of mature oocytes retrieved, clinical pregnancy rates and live birth rates in patients with a normal or poor ovarian reserve compared with follitropin alfa. Our study revealed better results with the dual stim protocol and the use of corifollitropin alfa, but more studies should be carried out on a larger group of patients to establish a clear correlation. Caution should be used when offering it in high responder patients because of the higher risk of hyperstimulation syndrome, but this is almost never the case when dealing with endometriosis [30,31,32,33].

The presence of endometriomas and particularly ovarian cystectomy for endometrioma have a negative impact on the number of retrieved oocytes [34,35]. In a group of patients with unoperated ovarian endometriotic cysts, ovarian stimulation resulted in a lower number of follicles and retrieved oocytes from the affected ovary as opposed to the healthy contralateral ovary [36]. In the case of ovarian cystectomy, a 2015 meta-analysis revealed that fewer oocytes were obtained from the operated ovary compared to the contralateral healthy ovary [37]. These studies are, however, contradictory to our findings, as we obtained no significant difference in the number of mature oocytes retrieved from the endometrioma group compared to the tubal factory group (5.28 vs. 4.02).

The clinical pregnancy rate in our study was 26.77% in the endometrioma group, which is much lower than 39.68% in the tubal obstruction group. Although we obtained quite similar stimulation results—average number of mature oocytes (5.28 vs. 4.02) and number of embryos (3.93 vs. 3.37)—we observed different results when it came to biochemical and clinical pregnancy rates, although both groups of patients had a similar average AMH level (1.54 ng/mL vs. 1.47 ng/mL). This conflicting outcome may be due to other deleterious effects of endometriosis on fertility other than those affecting the ovarian reserve: altered oocyte quality, chronic inflammatory peritoneal fluid with increased reactive oxygen species, progesterone resistance, anti-endometrial antibodies and luteal phase defects [38].

Most studies reveal conflicting data regarding the outcome of IVF in endometriosis. While some have only obtained lower fertilization rates and no difference in pregnancy rates between women with and without endometriosis, a large number of them reveal a lower average number of retrieved oocytes and lower pregnancy rates in endometriosis. A 2021 study comparing 862 women with endometrioma to a control group of women with other causes of infertility obtained a lower live birth rate attributable to endometrioma (39.32% vs. 46.87%). A better pregnancy rate in this study compared to our patients may be related to the higher average AMH level (3.02 ng/mL) [39,40,41,42,43].

In our study, we also encountered a higher fertilization failure in the endometrioma group (4.10%) than in the tubal obstruction group (1.47%) in the absence of a severely altered semen analysis, which indirectly explains the poor quality of the oocytes.

This study has its limitations as it is retrospective, and it shows the results of a single fertility center. Two particular limitations are that we did not take into account the male factor of infertility and embryo quality as a possible cause of implantation failure. Regarding male factor infertility, we did not include cases with teratospermia to minimize the chances of fertilization failure attributed to it. Regarding embryo quality, we considered that it indirectly reflects the quality of the oocyte, so we included all types of embryos obtained.

There is an endless discussion regarding the probability of endometriosis recurrence, estrogen-related cancers due to gonadotropin administration or the risk of endometriosis-associated cancer. A 2019 systematic review including 12 observational studies concluded that ovarian stimulation for IVF does not increase the progression or risk of endometriosis recurrence. Regarding the cancer risk, studies show conflicting results, but there is no increased risk of cancer in general. Recent meta-analyses from 2021 and 2022 show a small increased risk (+0.5–1.2%) of ovarian, breast and thyroid cancer and no correlation with cervical or colorectal cancer in patients with endometriosis. However, no specific cancer screening guidelines are implemented in this category of patients [44,45,46].

Considering the results of our study, but also taking into account its limitations, we thought that a future direction of research should focus on the embryological field. A study looking at pregnancy rates in relation to oocyte and embryo quality could be useful in terms of predicting reproductive outcome and also in providing patients a better understanding of the impact of endometriosis on their fertility. As there is currently no known treatment to improve oocyte quality, the focus should be on ovarian stimulation protocols and especially gonadotropins. From this point of view, more research should be carried out involving the luteal phase and the dual stim protocol, especially in patients with low ovarian reserve, and in terms of gonadotropins, corifolitropin has shown promising results, but more studies should be carried out.

## 5. Conclusions

Patients with a history of endometrioma are a special category of poor ovarian responders, and they are less likely to achieve pregnancy than those with tubal factor infertility, despite having a similar ovarian reserve and similar stimulation results regarding the number of oocytes and embryos. This negative outcome is further accentuated with advancing age.

Assisted reproductive technology has significantly increased the chances of patients with endometriosis to achieve pregnancy and are an option, sometimes the only option for those patients with advanced disease, where the spontaneous pregnancy rate is extremely low. Individualization of care is essential when dealing with endometriosis and infertility in order to optimize IVF outcomes, with ovarian stimulation protocol and gonadotropin type being the only few modifiable factors in the IVF process, along with luteal phase support treatment. Regarding the particularities of ovarian stimulation, both the short and the long protocol gave the same results in terms of pregnancy rate, but the dual trigger for final oocyte maturation did not lead to a higher pregnancy rate. The dual stim protocol and the use of corifollitropin alfa resulted in a better reproductive outcome for the endometrioma group, but more studies should be carried out on larger groups of patients to be able to draw a conclusion.

Although frequent, endometriosis still remains underdiagnosed and undertreated and is a great challenge for fertility specialists in terms of reproductive outcome and personalization of treatment.

## Figures and Tables

**Table 1:** Clinical characteristics, ovarian stimulation and reproductive outcome of the studied groups.

Clinical Characteristic	Endometrioma (Group A = 146) No (%)	Tubal Obstruction (Group B = 136) No (%)
Age (mean)	24–40 y (34 y)	24–40 y (37 y)
<35 y (%)	70 (47.94%)	53 (38.97%)
>35 y (%)	76 (52.05%)	83 (61.02%)
AMH ng/mL (mean)	0.08–4.46 ng/mL (1.54)	0.08–5.63 ng/mL (1.47)
<1 ng/mL	58 (39.72%)	72 (52.94%)
1–2 ng/mL	54 (36.98%)	41 (30.14%)
2–3 ng/mL	27 (18.49%)	18 (13.23%)
3 ng/mL	7 (4.79%)	5 (3.67%)
BMI kg/m^2^ (mean)	17.96–30.11 (22.54)	21.46–34.29 (27.36)
Mean age at menarche	12.93 y	13.28 y
Smoking	39 (26.71%)	52 (38.23%)
Dysmenorrhea	112 (76.71%)	76 (55.88%)
Dyspareunia	39 (26.71%)	12 (8.82%)
Chronic pelvic pain	51 (34.93%)	26 (19.11%)
Primary infertility	103 (70.54%)	72 (52.94%)
Previous ectopic pregnancy	8 (5.47%)	29 (21.32%)
Previous miscarriage	38 (26.02%)	62 (45.58%)
Ovarian stimulation and reproductive outcome		
Short protocol	123 (84.24%)	110 (80.88%)
Long protocol	18 (12.32%)	21 (15.44%)
Dual Stim	5 (3.42%)	5 (3.67%)
Dual trigger	65 (44.52%)	38 (27.94%)
Mean no of stimulation days	12.76 days	9.47 days
Mean no of mature oocytes	5.28	4.02
Mean no of embryos	3.93	3.37
Biochemical pregnancy rate	42.51%	46.82%
Clinical pregnancy rate	26.77%	39.68%

**Table 2:** Patients who achieved a clinical pregnancy in relation to the whole group.

	Endometrioma (*n* = 34)	Tubal Obstruction (*n* = 50)
Age		
<35 y (%)	23/70 (32.85%)	32/53 (60.37%)
>35 y (%)	11/76 (14.47%)	18/83 (21.68%)
Mean value of age	32.79 y	35.90 y
Ovarian stimulation protocol		
Short Protocol	28/123 (22.76%)	41/110 (37.27%)
Long Protocol	4/18 (22.22%)	6/21 (28.57%)
Dual Stim	2/5 (40%)	3/5 (60%)
Standard IVF	10/36 (27.77%)	23/60 (38.33%)
ICSI	24/100 (24%)	27/68 (39.70%)
Dual trigger	10/65 (15.38%)	12/38 (31.57%)
Embryo transfer		
Fresh ET	9/45 (20%)	29/77 (37.66%)
Frozen ET	25/82 (30.48%)	21/49 (42.85%)

## Data Availability

The data relevant to this study are presented in this article.
